# Supplementary material for: Association between single nucleotide polymorphisms (SNPs) of IL1, IL12, IL28 and TLR4 and symptoms of congenital cytomegalovirus infection
Source: PLoS One. 2020 May 18;15(5):e0233096. doi: 10.1371/journal.pone.0233096 (PMC7233583; doi:10.1371/journal.pone.0233096)
Supplement: S1 Table — Data presented as number (%), OR, odds ratio; CI, confidence interval; NA, not applicable; NS, not significant (p-values above 0.05); MRI, magnetic resonance imaging; IL, Interleukin; CCL 2, C-C motif chemokine ligand 2; DC-SIGN, dendritic cell-specific ICAM-grabbing non-integrin; TLR, Toll-like receptor. a SNP database (dbSNP) reference number (ID number). b P-value for comparison between infants with normal MRI and abnormal MRI in cCMV group. (DOCX) [file pone.0233096.s001.docx]

**Table S1. Association between examined SNPs and abnormal MRI.**

| **Gene** | **dbSNP IDnumber^a^** | **Genetic Model** | **Genotype** | **Normal MRI n=12** | **Abnormal MRI n=75** | **OR (95% CI)** | **P-value^b^** |
| --- | --- | --- | --- | --- | --- | --- | --- |
| **IL1B**  **G/A** | **rs16944** | **Codominant** | G/G | 5(41.7) | 30(40) | 1.00 | NS |
|  |  |  | A/G | 5(41.7) | 40(53.3) | 1.33(0.35-5.03) |  |
|  |  |  | A/A | 2(16.7) | 5(6.7) | 0.42(0.06-2.77) |  |
|  |  | **Dominant** | G/G | 5(41.7) | 30(40.0) | 1.00 | NS |
|  |  |  | A/G-A/A | 7(58.3) | 45(60.0) | 1.07(0.31-3.69) |  |
|  |  | **Recessive** | G/G-A/G | 10(83.3) | 70(93.3) | 1.00 | NS |
|  |  |  | A/A | 2(16.7) | 5(6.7) | 0.36(0.06-2.09) |  |
|  |  | **Overdominant** | G/G-A/A | 7(58.3) | 35(46.7) | 1.00 | NS |
|  |  |  | A/G | 5(41.7) | 40(53.3) | 1.60(0.47-5.50) |  |
|  |  | **Log-additive** | --- | --- | --- | 0.81(0.30-2.14) | NS |
| **IL12B**  **G/T** | **rs3212227** | **Codominant** | T/T | 8(66.7) | 45(60.0) | 1.00 | NS |
|  |  |  | T/G | 3(25) | 24(32.0) | 1.42(0.35-5.86) |  |
|  |  |  | G/G | 1(8.3) | 6(8.0) | 1.07(0.11-10.09) |  |
|  |  | **Dominant** | T/T | 8(66.7) | 45(60.0) | 1.00 | NS |
|  |  |  | T/G-G/G | 4(33.3) | 30(40.0) | 1.33(0.37-4.82) |  |
|  |  | **Recessive** | T/T-T/G | 11(91.7) | 69(92.0) | 1.00 | NS |
|  |  |  | G/G | 1(8.3) | 6(8.0) | 0.96(0.10-8.72) |  |
|  |  | **Overdominant** | T/T-G/G | 9(75.0) | 51(68.0) | 1.00 | NS |
|  |  |  | T/G | 3(25.0) | 24(32.0) | 1.41(0.35-5.69) |  |
|  |  | **Log-additive** | --- | --- | --- | 1.17(0.44-3.17) | NS |
| **IL28B**  **C/T** | **rs12979860** | **Codominant** | C/C | 7(58.3) | 30(40.0) | 1.00 | NS |
|  |  |  | T/C | 3(25) | 34(45.3) | 2.64(0.63-11.15) |  |
|  |  |  | T/T | 2(16.7) | 11(14.7) | 1.28(0.23-7.14) |  |
|  |  | **Dominant** | C/C | 7(58.3) | 30 (40) | 1.00 | NS |
|  |  |  | T/C-T/T | 5(41.7) | 45(60.0) | 2.10(0.61-7.24) |  |
|  |  | **Recessive** | C/C-T/C | 10(83.3) | 64(85.3) | 1.00 | NS |
|  |  |  | T/T | 2(16.7) | 11(14.7) | 0.86(0.17-4.46) |  |
|  |  | **Overdominant** | C/C-T/T | 9(75.0) | 41(54.7) | 1.00 | NS |
|  |  |  | T/C | 3(25.0) | 34(45.3) | 2.49(0.62-9.92) |  |
|  |  | **Log-additive** | --- | --- | --- | 1.41(0.57-3.51) | NS |
| **CCL2**  **A/G** | **rs1024611** | **Codominant** | A/A | 6(50.0) | 39(52.0) | 1.00 | NS |
|  |  |  | G/A | 6(50.0) | 33(44.0) | 0.85(0.25-2.87) |  |
|  |  |  | G/G | 0(0.0) | 3(4.0) | NA(0.00-NA) |  |
|  |  | **Dominant** | A/A | 6(50.0) | 39(52.0) | 1.00 | NS |
|  |  |  | G/A-G/G | 6(50.0) | 36(48.0) | 0.92(0.27-3.12) |  |
|  |  | **Recessive** | A/A-G/A | 12(100.0) | 72(96.0) | 1.00 | NS |
|  |  |  | G/G | 0(0.0) | 3(4.0) | NA(0.00-NA) |  |
|  |  | **Overdominant** | A/A-G/G | 6(50.0) | 42(56.0) | 1.00 | NS |
|  |  |  | G/A | 6(50.0) | 33(44.0) | 0.79(0.23-2.66) |  |
|  |  | **Log-additive** | --- | --- | --- | 1.07(0.36-3.16) | NS |
| **DC-SIGN**  **A/G** | **rs735240** | **Codominant** | G/G | 4(33.3) | 27(36.0) | 1.00 | NS |
|  |  |  | G/A | 5(41.7) | 31(41.3) | 0.92(0.22-3.77) |  |
|  |  |  | A/A | 3(25.0) | 17(22.7) | 0.84(0.17-4.22) |  |
|  |  | **Dominant** | G/G | 4(33.3) | 27(36.0) | 1.00 | NS |
|  |  |  | G/A-A/A | 8(66.7) | 48(64.0) | 0.89(0.24-3.23) |  |
|  |  | **Recessive** | G/G-G/A | 9(75.0) | 58(77.3) | 1.00 | NS |
|  |  |  | A/A | 3(25.0) | 17(22.7) | 0.88(0.21-3.62) |  |
|  |  | **Overdominant** | G/G-A/A | 7(58.3) | 44(58.7) | 1.00 | NS |
|  |  |  | G/A | 5(41.7) | 31(41.3) | 0.99(0.29-3.40) |  |
|  |  | **Log-additive** | --- | --- | --- | 0.92(0.41-2.05) | NS |
| **TLR2**  **A/G** | **rs5743708** | **---** | G/G | 11(91.7) | 67(89.3) | 1.00 | NS |
|  |  |  | G/A | 1(8.3) | 8(10.7) | 1.31(0.15-11.55) |  |
| **TLR4**  **C/T** | **rs4986791** | **---** | C/C | 12(100.0) | 67(89.3) | 1.00 | NS |
|  |  |  | T/C | 0(0.0) | 8(10.7) | NA(0.00-NA) |  |
| **TLR9**  **C/T** | **rs352140** | **Codominant** | T/T | 4(33.3) | 24(32.0) | 1.00 | NS |
|  |  |  | T/C | 6(50.0) | 38(50.7) | 1.06(0.27-4.13) |  |
|  |  |  | C/C | 2(16.7) | 13(17.3) | 1.08(0.17-6.73) |  |
|  |  | **Dominant** | T/T | 4(33.3) | 24(32.0) | 1.00 | NS |
|  |  |  | T/C-C/C | 8(66.7) | 51(68.0) | 1.06(0.29-3.88) |  |
|  |  | **Recessive** | T/T-T/C | 10(83.3) | 62(82.7) | 1.00 | NS |
|  |  |  | C/C | 2(16.7) | 13(17.3) | 1.05(0.21-5.36) |  |
|  |  | **Overdominant** | T/T-C/C | 6(50.0) | 37(49.3) | 1.00 | NS |
|  |  |  | T/C | 6(50.0) | 38(50.7) | 1.03(0.30-3.47) |  |
|  |  | **Log-additive** | --- | --- | --- | 1.04(0.43-2.54) | NS |

Data presented as number (%), OR, odds ratio; CI, confidence interval; NA, not applicable; NS, not significant (p-values above 0.05); MRI, magnetic resonance imaging; IL, Interleukin; CCL 2, C-C motif chemokine ligand 2; DC-SIGN, dendritic cell-specific ICAM-grabbing non-integrin; TLR, Toll-like receptor.
^a^ SNP database (dbSNP) reference number (ID number).

^b^ p-value for comparison between infants with normal MRI and abnormal MRI in cCMV group.
