# Supplementary material for: Association between single nucleotide polymorphisms (SNPs) of IL1, IL12, IL28 and TLR4 and symptoms of congenital cytomegalovirus infection
Source: PLoS One. 2020 May 18;15(5):e0233096. doi: 10.1371/journal.pone.0233096 (PMC7233583; doi:10.1371/journal.pone.0233096)
Supplement: S2 Table — Data presented as number (%), OR, odds ratio; CI, confidence interval; NA, not applicable; NS, not significant (p-values above 0.05); IL, Interleukin; CCL 2, C-C motif chemokine ligand 2; DC-SIGN, dendritic cell-specific ICAM-grabbing non-integrin; TLR, Toll-like receptor. a SNP database (dbSNP) reference number (ID number). b P-value for comparison between infants with normal hearing and abnormal hearing in cCMV group. c Abnormal hearing–was defined as air conduction thresholds > 20dBHL on the Auditory Brainstem Response (ABR) at least in one ear. (DOCX) [file pone.0233096.s002.docx]

**Table S2. Association between examined SNPs and abnormal hearing.**

| **Gene** | **dbSNP IDnumber^a^** | **Genetic Model** | **Genotype** | **Normal hearing n=63** | **Abnormal hearing^c^ n=29** | **OR (95% CI)** | **P-value^b^** |
| --- | --- | --- | --- | --- | --- | --- | --- |
| **IL1B**  **G/A** | **rs16944** | **Codominant** | G/G | 22(34.9) | 14(48.3) | 1.00 | NS |
|  |  |  | A/G | 36(57.1) | 13(44.8) | 0.57(0.23-1.43) |  |
|  |  |  | A/A | 5(7.9) | 2(6.9) | 0.63(0.11-3.70) |  |
|  |  | **Dominant** | G/G | 22(34.9) | 14(48.3) | 1.00 | NS |
|  |  |  | A/G-A/A | 41(65.1) | 15(51.7) | 0.57(0.24-1.41) |  |
|  |  | **Recessive** | G/G-A/G | 58(92.1) | 27(93.1) | 1.00 | NS |
|  |  |  | A/A | 5(7.9) | 2(6.9) | 0.86(0.16-4.71) |  |
|  |  | **Overdominant** | G/G-A/A | 27(42.9) | 16(55.2) | 1.00 | NS |
|  |  |  | A/G | 36(57.1) | 13(44.8) | 0.61(0.25-1.48) |  |
|  |  | **Log-additive** | --- | --- | --- | 0.67(0.32-1.41) | NS |
| **IL12B**  **G/T** | **rs3212227** | **Codominant** | T/T | 40(63.5) | 17(58.6) | 1.00 | NS |
|  |  |  | T/G | 18(28.6) | 10(34.5) | 1.31(0.50-3.41) |  |
|  |  |  | G/G | 5(7.9) | 2(6.9) | 0.94(0.17-5.34) |  |
|  |  | **Dominant** | T/T | 40(63.5) | 17(58.6) | 1.00 | NS |
|  |  |  | T/G-G/G | 23(36.5) | 12(41.4) | 1.23(0.50-3.02) |  |
|  |  | **Recessive** | T/T-T/G | 58(92.1) | 27(93.1) | 1.00 | NS |
|  |  |  | G/G | 5(7.9) | 2(6.9) | 0.86(0.16-4.71) |  |
|  |  | **Overdominant** | T/T-G/G | 45(71.4) | 19(65.5) | 1.00 | NS |
|  |  |  | T/G | 18(28.6) | 10(34.5) | 1.32(0.51-3.37) |  |
|  |  | **Log-additive** | --- | --- | --- | 1.10(0.55-2.19) | NS |
| **IL28B**  **C/T** | **rs12979860** | **Codominant** | C/C | 30(47.6) | 11(37.9) | 1.00 | NS |
|  |  |  | T/C | 24(38.1) | 14(48.3) | 1.59(0.61-4.13) |  |
|  |  |  | T/T | 9(14.3) | 4(13.8) | 1.21(0.31-4.75) |  |
|  |  | **Dominant** | C/C | 30(47.6) | 11(37.9) | 1.00 | NS |
|  |  |  | T/C-T/T | 33(52.4) | 18(62.1) | 1.49(0.61-3.65) |  |
|  |  | **Recessive** | C/C-T/C | 54(85.7) | 25(86.2) | 1.00 | NS |
|  |  |  | T/T | 9(14.3) | 4(13.8) | 0.96(0.27-3.42) |  |
|  |  | **Overdominant** | C/C-T/T | 39(61.9) | 15(51.7) | 1.00 | NS |
|  |  |  | T/C | 24(38.1) | 14(48.3) | 1.52(0.62-3.69) |  |
|  |  | **Log-additive** | --- | --- | --- | 1.20(0.65-2.24) | NS |
| **CCL2**  **A/G** | **rs1024611** | **Codominant** | A/A | 32(50.8) | 18(62.1) | 1.00 | NS |
|  |  |  | G/A | 29(46.0) | 10(34.5) | 0.61(0.24-1.54) |  |
|  |  |  | G/G | 2(3.2) | 1(3.5) | 0.89(0.08-10.50) |  |
|  |  | **Dominant** | A/A | 32(50.8) | 18(62.1) | 1.00 | NS |
|  |  |  | G/A-G/G | 31(49.2) | 11(37.9) | 0.63(0.26-1.55) |  |
|  |  | **Recessive** | A/A-G/A | 61(96.8) | 28(96.5) | 1.00 | NS |
|  |  |  | G/G | 2(3.2) | 1(3.5) | 1.09(0.09-12.52) |  |
|  |  | **Overdominant** | A/A-G/G | 34(54) | 19(65.5) | 1.00 | NS |
|  |  |  | G/A | 29(46) | 10(34.5) | 0.62(0.25-1.54) |  |
|  |  | **Log-additive** | --- | --- | --- | 0.70(0.31-1.57) | NS |
| **DC-SIGN**  **A/G** | **rs735240** | **Codominant** | G/G | 24(38.1) | 11(37.9) | 1.00 | NS |
|  |  |  | G/A | 24(38.1) | 13(44.8) | 1.18(0.44-3.16) |  |
|  |  |  | A/A | 15(23.8) | 5(17.2) | 0.73(0.21-2.51) |  |
|  |  | **Dominant** | G/G | 24(38.1) | 11(37.9) | 1.00 | NS |
|  |  |  | G/A-A/A | 39(61.9) | 18(62.1) | 1.01(0.41-2.49) |  |
|  |  | **Recessive** | G/G-G/A | 48(76.2) | 24(82.8) | 1.00 | NS |
|  |  |  | A/A | 15(23.8) | 5(17.2) | 0.67(0.22-2.05) |  |
|  |  | **Overdominant** | G/G-A/A | 39(61.9) | 16(55.2) | 1.00 | NS |
|  |  |  | G/A | 24(38.1) | 13(44.8) | 1.32(0.54-3.22) |  |
|  |  | **Log-additive** | --- | --- | --- | 0.89(0.50-1.60) | NS |
| **TLR2**  **A/G** | **rs5743708** | **---** | G/G | 56(88.9) | 26(89.7) | 1.00 | NS |
|  |  |  | G/A | 7(11.1) | 3(10.3) | 0.92(0.22-3.86) |  |
| **TLR4**  **C/T** | **rs4986791** | **---** | C/C | 57(90.5) | 26(89.7) | 1.00 | NS |
|  |  |  | T/C | 6(9.5) | 3(10.3) | 1.10(0.25-4.73) |  |
| **TLR9**  **C/T** | **rs352140** | **Codominant** | T/T | 19(30.2) | 11(37.9) | 1.00 | NS |
|  |  |  | T/C | 32(50.8) | 15(51.7) | 0.81(0.31-2.12) |  |
|  |  |  | C/C | 12(19.1) | 3(10.3) | 0.43(0.10-1.87) |  |
|  |  | **Dominant** | T/T | 19(30.2) | 11(37.9) | 1.00 | NS |
|  |  |  | T/C-C/C | 44(69.8) | 18(62.1) | 0.71(0.28-1.78) |  |
|  |  | **Recessive** | T/T-T/C | 51(81.0) | 26(89.7) | 1.00 | NS |
|  |  |  | C/C | 12(19.1) | 3(10.3) | 0.49(0.13-1.89) |  |
|  |  | **Overdominant** | T/T-C/C | 31(49.2) | 14(48.3) | 1.00 | NS |
|  |  |  | T/C | 32(50.8) | 15(51.7) | 1.04(0.43-2.50) |  |
|  |  | **Log-additive** | --- | --- | --- | 0.70(0.36-1.35) | NS |

Data presented as number (%), OR, odds ratio; CI, confidence interval; NA, not applicable; NS, not significant (p-values above 0.05);IL, Interleukin; CCL 2,C-C motif chemokine ligand 2; DC-SIGN, dendritic cell-specific ICAM-grabbing non-integrin; TLR, Toll-like receptor.
^a^ SNP database (dbSNP) reference number (ID number).
^b^ P-value for comparison between infants with normal hearing and abnormal hearing in cCMV group.
^c^ Abnormal hearing is defined as air conduction thresholds > 20dBHL on the Auditory Brainstem Response (ABR) at least in one ear.
