# Supplementary material for: Association between single nucleotide polymorphisms (SNPs) of IL1, IL12, IL28 and TLR4 and symptoms of congenital cytomegalovirus infection
Source: PLoS One. 2020 May 18;15(5):e0233096. doi: 10.1371/journal.pone.0233096 (PMC7233583; doi:10.1371/journal.pone.0233096)
Supplement: S3 Table — Data presented as number (%), OR, odds ratio; CI, confidence interval; NA, not applicable; NS, not significant (p-values above 0.05); IL, Interleukin; CCL2, C-C motif chemokine ligand 2; DC-SIGN, dendritic cell-specific ICAM-grabbing non-integrin; TLR, Toll-like receptor. a SNP database (dbSNP) reference number (ID number). b P-value for comparison between infants without cholestasis and with cholestasis in cCMV group. (DOCX) [file pone.0233096.s003.docx]

**Table S3. Association between examined SNPs and cholestasis.**

| **Gene** | **dbSNP IDnumber^a^** | **Genetic Model** | **Genotype** | **Without cholestasis n=71** | **With**  **cholestasis n=15** | **OR (95% CI)** | **P-value^b^** |
| --- | --- | --- | --- | --- | --- | --- | --- |
| **IL1B**  **G/A** | **rs16944** | **Codominant** | G/G | 27(38.0) | 7(46.7) | 1.00 | NS |
|  |  |  | A/G | 37(52.1) | 8(53.3) | 0.83(0.27-2.58) |  |
|  |  |  | A/A | 7(9.9) | 0(0.0) | 0.00(0.00-NA) |  |
|  |  | **Dominant** | G/G | 27(38) | 7(46.7) | 1.00 | NS |
|  |  |  | A/G-A/A | 44(62.0) | 8(53.3) | 0.70(0.23-2.15) |  |
|  |  | **Recessive** | G/G-A/G | 64(90.1) | 15(100.0) | 1.00 | NS |
|  |  |  | A/A | 7(9.9) | 0(0.0) | 0.00(0.00-NA) |  |
|  |  | **Overdominant** | G/G-A/A | 34(47.9) | 7(46.7) | 1.00 | NS |
|  |  |  | A/G | 37(52.1) | 8(53.3) | 1.05(0.34-3.21) |  |
|  |  | **Log-additive** | --- | --- | --- | 0.60(0.23-1.56) | NS |
| **IL12B**  **G/T** | **rs3212227** | **Codominant** | T/T | 45(63.4) | 9(60.0) | 1.00 | NS |
|  |  |  | T/G | 21(29.6) | 4(26.7) | 0.95(0.26-3.45) |  |
|  |  |  | G/G | 5(7.0) | 2(13.3) | 2.00(0.33-11.97) |  |
|  |  | **Dominant** | T/T | 45(63.4) | 9(60.0) | 1.00 | NS |
|  |  |  | T/G-G/G | 26(36.6) | 6(40.0) | 1.15(0.37-3.61) |  |
|  |  | **Recessive** | T/T-T/G | 66(93.0) | 13(86.7) | 1.00 | NS |
|  |  |  | G/G | 5(7.0) | 2(13.3) | 2.03(0.35-11.62) |  |
|  |  | **Overdominant** | T/T-G/G | 50(70.4) | 11(73.3) | 1.00 | NS |
|  |  |  | T/G | 21(29.6) | 4(26.7) | 0.87(0.25-3.03) |  |
|  |  | **Log-additive** | --- | --- | --- | 1.25(0.55-2.88) | NS |
| **IL28B**  **C/T** | **rs12979860** | **Codominant** | C/C | 34(47.9) | 4(26.7) | 1.00 | NS |
|  |  |  | T/C | 27(38.0) | 8(53.3) | 2.52(0.68-9.26) |  |
|  |  |  | T/T | 10(14.1) | 3(20.0) | 2.55(0.49-13.34) |  |
|  |  | **Dominant** | C/C | 34(47.9) | 4(26.7) | 1.00 | NS |
|  |  |  | T/C-T/T | 37(52.1) | 11(73.3) | 2.53(0.73-8.69) |  |
|  |  | **Recessive** | C/C-T/C | 61(85.9) | 12(80.0) | 1.00 | NS |
|  |  |  | T/T | 10(14.1) | 3(20.0) | 1.53(0.36-6.38) |  |
|  |  | **Overdominant** | C/C-T/T | 44(62.0) | 7(46.7) | 1.00 | NS |
|  |  |  | T/C | 27(38.0) | 8(53.3) | 1.86(0.61-5.72) |  |
|  |  | **Log-additive** | --- | --- | --- | 1.68(0.78-3.60) | NS |
| **CCL2**  **A/G** | **rs1024611** | **Codominant** | A/A | 37(52.1) | 9(60.0) | 1.00 | NS |
|  |  |  | G/A | 32(45.1) | 5(33.3) | 0.64(0.20-2.11) |  |
|  |  |  | G/G | 2(2.8) | 1(6.7) | 2.06(0.17-25.26) |  |
|  |  | **Dominant** | A/A | 37(52.1) | 9(60.0) | 1.00 | NS |
|  |  |  | G/A-G/G | 34(47.9) | 6(40.0) | 0.73(0.23-2.25) |  |
|  |  | **Recessive** | A/A-G/A | 69(97.2) | 14(93.3) | 1.00 | NS |
|  |  |  | G/G | 2(2.8) | 1(6.7) | 2.46(0.21-29.08) |  |
|  |  | **Overdominant** | A/A-G/G | 39(54.9) | 10(66.7) | 1.00 | NS |
|  |  |  | G/A | 32(45.1) | 5(33.3) | 0.61(0.19-1.97) |  |
|  |  | **Log-additive** | --- | --- | --- | 0.88(0.32-2.39) | NS |
| **DC-SIGN**  **A/G** | **rs735240** | **Codominant** | G/G | 27(38) | 6(40.0) | 1.00 | NS |
|  |  |  | G/A | 29(40.9) | 6(40.0) | 0.93(0.27-3.24) |  |
|  |  |  | A/A | 15(21.1) | 3(20.0) | 0.90(0.20-4.13) |  |
|  |  | **Dominant** | G/G | 27(38.0) | 6(40.0) | 1.00 | NS |
|  |  |  | G/A-A/A | 44(62.0) | 9(60.0) | 0.92(0.29-2.87) |  |
|  |  | **Recessive** | G/G-G/A | 56(78.9) | 12(80.0) | 1.00 | NS |
|  |  |  | A/A | 15(21.1) | 3(20.0) | 0.93(0.23-3.74) |  |
|  |  | **Overdominant** | G/G-A/A | 42(59.1) | 9(60.0) | 1.00 | NS |
|  |  |  | G/A | 29(40.9) | 6(40.0) | 0.97(0.31-3.01) |  |
|  |  | **Log-additive** | --- | --- | --- | 0.95(0.45-1.99) | NS |
| **TLR2**  **A/G** | **rs5743708** | **---** | G/G | 62(87.3) | 15(100.0) | 1.00 | NS |
|  |  |  | G/A | 9(12.7) | 0(0.0) | 0.00(0.00-NA) |  |
| **TLR4**  **C/T** | **rs4986791** | **---** | C/C | 65(91.5) | 12(80.0) | 1.00 | NS |
|  |  |  | T/C | 6(8.4) | 3(20.0) | 2.71(0.59-12.34) |  |
| **TLR9**  **C/T** | **rs352140** | **Codominant** | T/T | 24(33.8) | 5(33.3) | 1.00 | NS |
|  |  |  | T/C | 37(52.1) | 7(46.7) | 0.91(0.26-3.19) |  |
|  |  |  | C/C | 10(14.1) | 3(20.0) | 1.44(0.29-7.21) |  |
|  |  | **Dominant** | T/T | 24(33.8) | 5(33.3) | 1.00 | NS |
|  |  |  | T/C-C/C | 47(66.2) | 10(66.7) | 1.02(0.31-3.33) |  |
|  |  | **Recessive** | T/T-T/C | 61(85.9) | 12(80.0) | 1.00 | NS |
|  |  |  | C/C | 10(14.1) | 3(20.0) | 1.53(0.36-6.38) |  |
|  |  | **Overdominant** | T/T-C/C | 34(47.9) | 8(53.3) | 1.00 | NS |
|  |  |  | T/C | 37(52.1) | 7(46.7) | 0.80(0.26-2.45) |  |
|  |  | **Log-additive** | --- | --- | --- | 1.15(0.51-2.62) | NS |

Data presented as number (%), OR, odds ratio; CI, confidence interval; NA, not applicable; NS, not significant (p-values above 0.05); IL, Interleukin; CCL 2, C-C motif chemokine ligand 2; DC-SIGN, dendritic cell-specific ICAM-grabbing non-integrin; TLR, Toll-like receptor.
^a^ SNP database (dbSNP) reference number (ID number).

^b^ P-value for comparison between infants without cholestasis and with cholestasis in cCMV group.
