# Supplementary material for: Association between single nucleotide polymorphisms (SNPs) of IL1, IL12, IL28 and TLR4 and symptoms of congenital cytomegalovirus infection
Source: PLoS One. 2020 May 18;15(5):e0233096. doi: 10.1371/journal.pone.0233096 (PMC7233583; doi:10.1371/journal.pone.0233096)
Supplement: S4 Table — Data presented as number (%), OR, odds ratio; CI, confidence interval; NA, not applicable; NS, not significant (p-values above 0.05); IUGR, intrauterine growth restriction; IL, Interleukin; CCL 2, C-C motif chemokine ligand 2; DC-SIGN, dendritic cell-specific ICAM-grabbing non-integrin; TLR, Toll-like receptor.a SNP database (dbSNP) reference number (ID number). b P-value for comparison between infants without IUGR and with IUGR in cCMV group. (DOCX) [file pone.0233096.s004.docx]

**Table S4. Association between examined SNPs and IUGR.**

| **Gene** | **dbSNP IDnumber^a^** | **Genetic Model** | **Genotype** | **Without IUGR n=66** | **With**  **IUGR n=26** | **OR (95% CI)** | **P-value^b^** |
| --- | --- | --- | --- | --- | --- | --- | --- |
| **IL1B**  **G/A** | **rs16944** | **Codominant** | G/G | 29(43.9) | 7(26.9) | 1.00 | NS |
|  |  |  | A/G | 31(47.0) | 18(69.2) | 2.41(0.88-6.60) |  |
|  |  |  | A/A | 6(9.1) | 1(3.8) | 0.69(0.07-6.70) |  |
|  |  | **Dominant** | G/G | 29(43.9) | 7(26.9) | 1.00 | NS |
|  |  |  | A/G-A/A | 37(56.1) | 19(73.1) | 2.13(0.79-5.75) |  |
|  |  | **Recessive** | G/G-A/G | 60(90.9) | 25(96.2) | 1.00 | NS |
|  |  |  | A/A | 6(9.1) | 1(3.8) | 0.40(0.05-3.50) |  |
|  |  | **Overdominant** | G/G-A/A | 35(53.0) | 8(30.8) | 1.00 | NS |
|  |  |  | A/G | 31(47.0) | 18(69.2) | 2.54(0.97-6.65) |  |
|  |  | **Log-additive** | --- | --- | --- | 1.37(0.65-2.90) | NS |
| **IL12B**  **G/T** | **rs3212227** | **Codominant** | T/T | 43(65.2) | 14(53.9) | 1.00 | NS |
|  |  |  | T/G | 20(30.3) | 8(30.8) | 1.23(0.44-3.40) |  |
|  |  |  | G/G | 3(4.5) | 4(15.4) | 4.10(0.82-20.57) |  |
|  |  | **Dominant** | T/T | 43(65.2) | 14(53.9) | 1.00 | NS |
|  |  |  | T/G-G/G | 23(34.9) | 12(46.1) | 1.60(0.64-4.03) |  |
|  |  | **Recessive** | T/T-T/G | 63(95.5) | 22(84.6) | 1.00 | NS |
|  |  |  | G/G | 3(4.5) | (415.4) | 3.82(0.79-18.42) |  |
|  |  | **Overdominant** | T/T-G/G | 46(69.7) | 18(69.2) | 1.00 | NS |
|  |  |  | T/G | 20(30.3) | 8(30.8) | 1.02(0.38-2.74) |  |
|  |  | **Log-additive** | --- | --- | --- | 1.69(0.85-3.38) | NS |
| **IL28B**  **C/T** | **rs12979860** | **Codominant** | C/C | 32(48.5) | 9(34.6) | 1.00 | NS |
|  |  |  | T/C | 25(37.9) | 13(50.0) | 1.85(0.68-5.02) |  |
|  |  |  | T/T | 9(13.6) | 4(15.4) | 1.58(0.39-6.35) |  |
|  |  | **Dominant** | C/C | 32(48.5) | 9(34.6) | 1.00 | NS |
|  |  |  | T/C-T/T | 34(51.5) | 17(65.4) | 1.78(0.69-4.56) |  |
|  |  | **Recessive** | C/C-T/C | 57(86.4) | 22(84.6) | 1.00 | NS |
|  |  |  | T/T | 9(13.6) | 4(15.4) | 1.15(0.32-4.13) |  |
|  |  | **Overdominant** | C/C-T/T | 41(62.1) | 13(50.0) | 1.00 | NS |
|  |  |  | T/C | 25(37.9) | 13(50.0) | 1.64(0.66-4.10) |  |
|  |  | **Log-additive** | --- | --- | --- | 1.36(0.72-2.58) | NS |
| **CCL2**  **A/G** | **rs1024611** | **Codominant** | A/A | 37(56.1) | 13(50.0) | 1.00 | NS |
|  |  |  | G/A | 29(43.9) | 10(38.5) | 0.98(0.38-2.56) |  |
|  |  |  | G/G | 0(0.0) | 3(11.5) | NA(0.00-NA) |  |
|  |  | **Dominant** | A/A | 37(56.1) | 13(50.0) | 1.00 | NS |
|  |  |  | G/A-G/G | 29(43.9) | 13(50.0) | 1.28(0.51-3.17) |  |
|  |  | **Recessive** | A/A-G/A | 66(100.0) | 23(88.5) | 1.00 | NS |
|  |  |  | G/G | 0(0.0) | 3(11.5) | NA(0.00-NA) |  |
|  |  | **Overdominant** | A/A-G/G | 37(56.1) | 16(61.5) | 1.00 | NS |
|  |  |  | G/A | 29(43.9) | 10(38.5) | 0.80(0.32-2.02) |  |
|  |  | **Log-additive** | --- | --- | --- | 1.73(0.78-3.85) | NS |
| **DC-SIGN**  **A/G** | **rs735240** | **Codominant** | G/G | 24(36.4) | 11(42.3) | 1.00 | NS |
|  |  |  | G/A | 27(40.9) | 10(38.5) | 0.81(0.29-2.24) |  |
|  |  |  | A/A | 15(22.7) | (519.2) | 0.73(0.21-2.51) |  |
|  |  | **Dominant** | G/G | 24(36.4) | 11(42.3) | 1.00 | NS |
|  |  |  | G/A-A/A | 42(63.6) | 15(57.7) | 0.78(0.31-1.97) |  |
|  |  | **Recessive** | G/G-G/A | 51(77.3) | 21(80.8) | 1.00 | NS |
|  |  |  | A/A | 15(22.7) | 5(19.2) | 0.81(0.26-2.51) |  |
|  |  | **Overdominant** | G/G-A/A | 39(59.1) | 16(61.5) | 1.00 | NS |
|  |  |  | G/A | 27(40.9) | 10(38.5) | 0.90(0.36-2.29) |  |
|  |  | **Log-additive** | --- | --- | --- | 0.85(0.46-1.55) | NS |
| **TLR2**  **A/G** | **rs5743708** | **---** | G/G | 60(90.9) | 22(84.6) | 1.00 | NS |
|  |  |  | G/A | 6(9.1) | 4(15.4) | 1.82(0.47-7.06) |  |
| **TLR4**  **C/T** | **rs4986791** | **---** | C/C | 60(90.9) | 23(88.5) | 1.00 | NS |
|  |  |  | T/C | 6(9.1) | 3(11.5) | 1.30(0.30-5.66) |  |
| **TLR9**  **C/T** | **rs352140** | **Codominant** | T/T | 20(30.3) | 10(38.5) | 1.00 | NS |
|  |  |  | T/C | 33(50.0) | 14(53.9) | 0.85(0.32-2.27) |  |
|  |  |  | C/C | 13(19.7) | 2(7.7) | 0.31(0.06-1.64) |  |
|  |  | **Dominant** | T/T | 20(30.3) | 10(38.5) | 1.00 | NS |
|  |  |  | T/C-C/C | 46(69.7) | 16(61.5) | 0.70(0.27-1.80) |  |
|  |  | **Recessive** | T/T-T/C | 53(80.3) | 24(92.3) | 1.00 | NS |
|  |  |  | C/C | 13(19.7) | 2(7.7) | 0.34(0.07-1.62) |  |
|  |  | **Overdominant** | T/T-C/C | 33(50.0) | 12(46.1) | 1.00 | NS |
|  |  |  | T/C | 33(50.0) | 14(53.9) | 1.17(0.47-2.90) |  |
|  |  | **Log-additive** | --- | --- | --- | 0.64(0.32-1.28) | NS |

Data presented as number (%), OR, odds ratio; CI, confidence interval; NA, not applicable; NS, not significant (p-values above 0.05); IUGR, intrauterine growth restriction; IL, Interleukin; CCL 2,C-C motif chemokine ligand 2; DC-SIGN, dendritic cell-specific ICAM-grabbing non-integrin; TLR, Toll-like receptor.
^a^ SNP database (dbSNP) reference number (ID number).

^b^ P-value for comparison between infants without IUGR and with IUGR in cCMV group.
