# Supplementary material for: Association between single nucleotide polymorphisms (SNPs) of IL1, IL12, IL28 and TLR4 and symptoms of congenital cytomegalovirus infection
Source: PLoS One. 2020 May 18;15(5):e0233096. doi: 10.1371/journal.pone.0233096 (PMC7233583; doi:10.1371/journal.pone.0233096)
Supplement: S5 Table — Data presented as number (%), OR, odds ratio; CI, confidence interval; NA, not applicable; NS, not significant (p-values above 0.05); IL, Interleukin; CCL 2, C-C motif chemokine ligand 2; DC-SIGN, dendritic cell-specific ICAM-grabbing non-integrin; TLR, Toll-like receptor. a SNP database (dbSNP) reference number (ID number). b P-value for comparison between infants without microcephaly and with microcephaly in cCMV group. (DOCX) [file pone.0233096.s005.docx]

**Table S5. Association between examined SNPs and microcephaly.**

| **Gene** | **dbSNP IDnumber^a^** | **Genetic Model** | **Genotype** | **Without microcephaly n=70** | **With**  **microcephaly n=22** | **OR (95% CI)** | **P-value^b^** |
| --- | --- | --- | --- | --- | --- | --- | --- |
| **IL1B**  **G/A** | **rs16944** | **Codominant** | G/G | 28(40.0) | 8(36.4) | 1.00 | NS |
|  |  |  | A/G | 37(52.9) | 12(54.5) | 1.14(0.41-3.15) |  |
|  |  |  | A/A | 5(7.1) | 2(9.1) | 1.40(0.23-8.63) |  |
|  |  | **Dominant** | G/G | 28(40.0) | 8(36.4) | 1.00 | NS |
|  |  |  | A/G-A/A | 42(60.0) | 14(63.6) | 1.17(0.43-3.14) |  |
|  |  | **Recessive** | G/G-A/G | 65(92.9) | 20(90.9) | 1.00 | NS |
|  |  |  | A/A | 5(7.1) | 2(9.1) | 1.30(0.23-7.22) |  |
|  |  | **Overdominant** | G/G-A/A | 33(47.1) | 10(45.5) | 1.00 | NS |
|  |  |  | A/G | 37(52.9) | 12(54.5) | 1.07(0.41-2.80) |  |
|  |  | **Log-additive** | --- | --- | --- | 1.16(0.53-2.55) | NS |
| **IL12B**  **G/T** | **rs3212227** | **Codominant** | T/T | 44(62.9) | 13(59.1) | 1.00 | NS |
|  |  |  | T/G | 21(30) | 7(31.8) | 1.13(0.39-3.24) |  |
|  |  |  | G/G | 5(7.1) | 2(9.1) | 1.35(0.23-7.81) |  |
|  |  | **Dominant** | T/T | 44(62.9) | 13(59.1) | 1.00 | NS |
|  |  |  | T/G-G/G | 26(37.1) | 9(40.9) | 1.17(0.44-3.12) |  |
|  |  | **Recessive** | T/T-T/G | 65(92.9) | 20(90.9) | 1.00 | NS |
|  |  |  | G/G | 5(7.1) | 2(9.1) | 1.30(0.23-7.22) |  |
|  |  | **Overdominant** | T/T-G/G | 49(70.0) | 15(68.2) | 1.00 | NS |
|  |  |  | T/G | 21(30.0) | 7(31.8) | 1.09(0.39-3.06) |  |
|  |  | **Log-additive** | --- | --- | --- | 1.15(0.55-2.41) | NS |
| **IL28B**  **C/T** | **rs12979860** | **Codominant** | C/C | 31(44.3) | 10(45.5) | 1.00 | NS |
|  |  |  | T/C | 27(38.6) | 11(50) | 1.26(0.46-3.43) |  |
|  |  |  | T/T | 12(17.1) | 1(4.5) | .26(0.03-2.24) |  |
|  |  | **Dominant** | C/C | 31(44.3) | 10(45.5) | 1.00 | NS |
|  |  |  | T/C-T/T | 39(55.7) | 12(54.5) | 0.95(0.36-2.50) |  |
|  |  | **Recessive** | C/C-T/C | 58(82.9) | 21(95.5) | 1.00 | NS |
|  |  |  | T/T | 12(17.1) | 1(4.5) | 0.23(0.03-1.88) |  |
|  |  | **Overdominant** | C/C-T/T | 43(61.4) | 11(50.0) | 1.00 | NS |
|  |  |  | T/C | 27(38.6) | 11(50.0) | 1.59(0.61-4.18) |  |
|  |  | **Log-additive** | --- | --- | --- | 0.75(0.37-1.52) | NS |
| **CCL2**  **A/G** | **rs1024611** | **Codominant** | A/A | 35(50.0) | 15(68.2) | 1.00 | NS |
|  |  |  | G/A | 33(47.1) | 6(27.3) | 0.42(0.15-1.22) |  |
|  |  |  | G/G | 2(2.9) | 1(4.5) | 1.17(0.10-13.87) |  |
|  |  | **Dominant** | A/A | 35(50.0) | 15(68.2) | 1.00 | NS |
|  |  |  | G/A-G/G | 35(50.0) | 7(31.8) | 0.47(0.17-1.28) |  |
|  |  | **Recessive** | A/A-G/A | 68(97.1) | 21(95.5) | 1.00 | NS |
|  |  |  | G/G | 2(2.9) | 1(4.5) | 1.62(0.14-18.76) |  |
|  |  | **Overdominant** | A/A-G/G | 37(52.9) | 16(72.7) | 1.00 | NS |
|  |  |  | G/A | 33(47.1) | 6(27.3) | 0.42(0.15-1.20) |  |
|  |  | **Log-additive** | --- | --- | --- | 0.57(0.23-1.43) | NS |
| **DC-SIGN**  **A/G** | **rs735240** | **Codominant** | G/G | 27(38.6) | 8(36.4) | 1.00 | NS |
|  |  |  | G/A | 27(38.6) | 10(45.5) | 1.25(0.43-3.65) |  |
|  |  |  | A/A | 16(22.9) | 4(18.2) | 0.84(0.22-3.26) |  |
|  |  | **Dominant** | G/G | 27(38.6) | 8(36.4) | 1.00 | NS |
|  |  |  | G/A-A/A | 43(61.4) | 14(63.6) | 1.10(0.41-2.97) |  |
|  |  | **Recessive** | G/G-G/A | 54(77.1) | 18(81.8) | 1.00 | NS |
|  |  |  | A/A | 16(22.9) | 4(18.2) | 0.75(0.22-2.54) |  |
|  |  | **Overdominant** | G/G-A/A | 43(61.4) | 12(54.5) | 1.00 | NS |
|  |  |  | G/A | 27(38.6) | 10(45.5) | 1.33(0.50-3.49) |  |
|  |  | **Log-additive** | --- | --- | --- | 0.96(0.51-1.81) | NS |
| **TLR2**  **A/G** | **rs5743708** | **---** | G/G | 64(91.4) | 18(81.8) | 1.00 | NS |
|  |  |  | G/A | 6(8.6) | 4(18.2) | 2.37(0.60-9.32) |  |
| **TLR4**  **C/T** | **rs4986791** | **---** | C/C | 64(91.4) | 19(86.4) | 1.00 | NS |
|  |  |  | T/C | 6(8.6) | 3(13.6) | 1.68(0.38-7.38) |  |
| **TLR9**  **C/T** | **rs352140** | **Codominant** | T/T | 23(32.9) | 7(31.8) | 1.00 | NS |
|  |  |  | T/C | 34(48.6) | 13(59.1) | 1.26(0.44-3.63) |  |
|  |  |  | C/C | 13(18.6) | 2(9.1) | 0.51(0.09-2.80) |  |
|  |  | **Dominant** | T/T | 23(32.9) | 7(31.8) | 1.00 | NS |
|  |  |  | T/C-C/C | 47(67.1) | 15(68.2) | 1.05(0.38-2.93) |  |
|  |  | **Recessive** | T/T-T/C | 57(81.4) | (90.9) | 1.00 | NS |
|  |  |  | C/C | 13(18.6) | (9.1) | 0.44(0.09-2.11) |  |
|  |  | **Overdominant** | T/T-C/C | 36(51.4) | 9(40.9) | 1.00 | NS |
|  |  |  | T/C | 34(48.6) | 13(59.1) | 1.53(0.58-4.04) |  |
|  |  | **Log-additive** | --- | --- | --- | 0.83(0.41-1.70) | NS |

Data presented as number (%), OR, odds ratio; CI, confidence interval; NA, not applicable; NS, not significant (p-values above 0.05); IL, Interleukin; CCL 2, C-C motif chemokine ligand 2; DC-SIGN, dendritic cell-specific ICAM-grabbing non-integrin; TLR, Toll-like receptor.
^a^ SNP database (dbSNP) reference number (ID number).

^b^ P-value for comparison between infants without microcephaly and with microcephaly in cCMV group.
