# Supplementary material for: Association between single nucleotide polymorphisms (SNPs) of IL1, IL12, IL28 and TLR4 and symptoms of congenital cytomegalovirus infection
Source: PLoS One. 2020 May 18;15(5):e0233096. doi: 10.1371/journal.pone.0233096 (PMC7233583; doi:10.1371/journal.pone.0233096)
Supplement: S6 Table — Data presented as number (%), OR, odds ratio; CI, confidence interval; NA, not applicable; NS, not significant (p-values above 0.05); IL, Interleukin; CCL 2, C-C motif chemokine ligand 2; DC-SIGN, dendritic cell-specific ICAM-grabbing non-integrin; TLR, Toll-like receptor. a SNP database (dbSNP) reference number (ID number). b P-value for comparison between infants without neutropenia and with neutropenia in cCMV group. (DOCX) [file pone.0233096.s006.docx]

**Table S6. Association between examined SNPs and neutropenia.**

| **Gene** | **dbSNP IDnumber^a^** | **Genetic Model** | **Genotype** | **Without neutropenia n=82** | **With**  **neutropenia n=10** | **OR (95% CI)** | **P-value^b^** |
| --- | --- | --- | --- | --- | --- | --- | --- |
| **IL1B**  **G/A** | **rs16944** | **Codominant** | G/G | 30(36.6) | 6(60.0) | 1.00 | NS |
|  |  |  | A/G | 46(56.1) | 3(30.0) | 0.33(0.08-1.40) |  |
|  |  |  | A/A | 6(7.3) | 1(10.0) | 0.83(0.08-8.24) |  |
|  |  | **Dominant** | G/G | 30(36.6) | 6(60.0) | 1.00 | NS |
|  |  |  | A/G-A/A | 52(63.4) | 4(40.0) | 0.38(0.10-1.47) |  |
|  |  | **Recessive** | G/G-A/G | 76(92.7) | 9(90.0) | 1.00 | NS |
|  |  |  | A/A | 6(7.3) | 1(10.0) | 1.41(0.15-13.05) |  |
|  |  | **Overdominant** | G/G-A/A | 36(43.9) | 7(70.0) | 1.00 | NS |
|  |  |  | A/G | 46(56.1) | 3(30.0) | 0.34(0.08-1.39) |  |
|  |  | **Log-additive** | --- | --- | --- | 0.55(0.17-1.75) | NS |
| **IL12B**  **G/T** | **rs3212227** | **Codominant** | T/T | 53(64.6) | 4(40.0) | 1.00 | NS |
|  |  |  | T/G | 24(29.3) | 4(40.0) | 2.21(0.51-9.58) |  |
|  |  |  | G/G | 5(6.1) | 2(20.0) | 5.30(0.77-36.49) |  |
|  |  | **Dominant** | T/T | 53(64.6) | 4(40.0) | 1.00 | NS |
|  |  |  | T/G-G/G | 29(35.4) | 6(60.0) | 2.74(0.72-10.51) |  |
|  |  | **Recessive** | T/T-T/G | 77(93.9) | 8(80.0) | 1.00 | NS |
|  |  |  | G/G | 5(6.1) | 2(20.0) | 3.85(0.64-23.16) |  |
|  |  | **Overdominant** | T/T-G/G | 58(70.7) | 6(60.0) | 1.00 | NS |
|  |  |  | T/G | 24(29.3) | 4(40.0) | 1.61(0.42-6.23) |  |
|  |  | **Log-additive** | --- | --- | --- | 2.28(0.91-5.75) | NS |
| **IL28B**  **C/T** | **rs12979860** | **Codominant** | C/C | 37(45.1) | 4(40.0) | 1.00 | NS |
|  |  |  | T/C | 33(40.2) | 5(50.0) | 1.40(0.35-5.66) |  |
|  |  |  | T/T | 12(14.6) | 1(10.0) | 0.77(0.08-7.58) |  |
|  |  | **Dominant** | C/C | 37(45.1) | 4(40.0) | 1.00 | NS |
|  |  |  | T/C-T/T | 45(54.9) | 6(60.0) | 1.23(0.32-4.70) |  |
|  |  | **Recessive** | C/C-T/C | 70(85.4) | 9(90.0) | 1.00 | NS |
|  |  |  | T/T | 12(14.6) | 1(10.0) | 0.65(0.08-5.59) |  |
|  |  | **Overdominant** | C/C-T/T | 49(59.8) | 5(50.0) | 1.00 | NS |
|  |  |  | T/C | 33(40.2) | 5(50.0) | 1.48(0.40-5.54) |  |
|  |  | **Log-additive** | --- | --- | --- | 1.01(0.40-2.57) | NS |
| **CCL2**  **A/G** | **rs1024611** | **Codominant** | A/A | 42(51.2) | 8(80.0) | 1.00 | NS |
|  |  |  | G/A | 37(45.1) | 2(20.0) | 0.28(0.06-1.42) |  |
|  |  |  | G/G | 3(3.7) | 0(0.0) | 0.00(0.00-NA) |  |
|  |  | **Dominant** | A/A | 42(51.2) | 8(80.0) | 1.00 | NS |
|  |  |  | G/A-G/G | 40(48.8) | 2(20.0) | 0.26(0.05-1.31) |  |
|  |  | **Recessive** | A/A-G/A | 79(96.3) | 10(100.0) | 1.00 | NS |
|  |  |  | G/G | 3(3.7) | 0(0.0) | 0.00(0.00-NA) |  |
|  |  | **Overdominant** | A/A-G/G | 45(54.9) | 8(80.0) | 1.00 | NS |
|  |  |  | G/A | 37(45.1) | 2(20.0) | 0.30(0.06-1.52) |  |
|  |  | **Log-additive** | --- | --- | --- | 0.27(0.06-1.29) | NS |
| **DC-SIGN**  **A/G** | **rs735240** | **Codominant** | G/G | 30(36.6) | 5(50.0) | 1.00 | NS |
|  |  |  | G/A | 34(41.5) | 3(30.0) | 0.53(0.12-2.40) |  |
|  |  |  | A/A | 18(21.9) | 2(20.0) | 0.67(0.12-3.80) |  |
|  |  | **Dominant** | G/G | 30(36.6) | 5(50.0) | 1.00 | NS |
|  |  |  | G/A-A/A | 52(63.4) | 5(50.0) | 0.58(0.15-2.16) |  |
|  |  | **Recessive** | G/G-G/A | 64(78.0) | 8(80.0) | 1.00 | NS |
|  |  |  | A/A | 18(21.9) | 2(20.0) | 0.89(0.17-4.56) |  |
|  |  | **Overdominant** | G/G-A/A | 48(58.5) | 7(70.0) | 1.00 | NS |
|  |  |  | G/A | 34(41.5) | 3(30.0) | 0.61(0.15-2.51) |  |
|  |  | **Log-additive** | --- | --- | --- | 0.76(0.31-1.86) | NS |
| **TLR2**  **A/G** | **rs5743708** | **---** | G/G | 72(87.8) | 10(100.0) | 1.00 | NS |
|  |  |  | G/A | 10(12.2) | 0 | 0.00(0.00-NA) |  |
| **TLR4**  **C/T** | **rs4986791** | **---** | C/C | 74(90.2) | 9(90.0) | 1.00 | NS |
|  |  |  | T/C | 8(9.8) | 1(10.0) | 1.03(0.11-9.19) |  |
| **TLR9**  **C/T** | **rs352140** | **Codominant** | T/T | 26(31.7) | 4(40.0) | 1.00 | NS |
|  |  |  | T/C | 43(52.4) | 4(40.0) | 0.60(0.14-2.63) |  |
|  |  |  | C/C | 13(15.8) | 2(20.0) | 1.00(0.16-6.19) |  |
|  |  | **Dominant** | T/T | 26(31.7) | 4(40.0) | 1.00 | NS |
|  |  |  | T/C-C/C | 56(68.3) | 6(60.0) | 0.70(0.18-2.68) |  |
|  |  | **Recessive** | T/T-T/C | 69(84.2) | 8(80.0) | 1.00 | NS |
|  |  |  | C/C | 13(15.8) | 2(20.0) | 1.33(0.25-6.97) |  |
|  |  | **Overdominant** | T/T-C/C | 39(47.6) | 6(60.0) | 1.00 | NS |
|  |  |  | T/C | 43(52.4) | 4(40.0) | 0.60(0.16-2.30) |  |
|  |  | **Log-additive** | --- | --- | --- | 0.91(0.35-2.41) | NS |

Data presented as number (%), OR, odds ratio; CI, confidence interval; NA, not applicable; NS, not significant (p-values above 0.05); IL, Interleukin; CCL 2,C-C motif chemokine ligand 2; DC-SIGN, dendritic cell-specific ICAM-grabbing non-integrin; TLR, Toll-like receptor.
^a^ SNP database (dbSNP) reference number (ID number).

^b^ P-value for comparison between infants without neutropenia and with neutropenia in cCMV group.
