# Supplementary material for: Association between single nucleotide polymorphisms (SNPs) of IL1, IL12, IL28 and TLR4 and symptoms of congenital cytomegalovirus infection
Source: PLoS One. 2020 May 18;15(5):e0233096. doi: 10.1371/journal.pone.0233096 (PMC7233583; doi:10.1371/journal.pone.0233096)
Supplement: S7 Table — Data presented as number (%), OR, odds ratio; CI, confidence interval; NA, not applicable; NS, not significant (p-values above 0.05); IL, Interleukin; CCL 2, C-C motif chemokine ligand 2; DC-SIGN, dendritic cell-specific ICAM-grabbing non-integrin; TLR, Toll-like receptor. a SNP database (dbSNP) reference number (ID number). b P-value for comparison between infants without chorioretinitis and with chorioretinitis in cCMV group. c With chorioretinitis–if occurred at least in one eye. (DOCX) [file pone.0233096.s007.docx]

**Table S7. Association between examined SNPs and chorioretinitis.**

| **Gene** | **dbSNP IDnumber^a^** | **Genetic Model** | **Genotype** | **Without**  **chorioretinitis n=75** | **With**  **chorioretinitis^c^ n=17** | **OR (95% CI)** | **P-value^b^** |
| --- | --- | --- | --- | --- | --- | --- | --- |
| **IL1B**  **G/A** | **rs16944** | **Codominant** | G/G | 30(40.0) | 6(35.3) | 1.00 | NS |
|  |  |  | A/G | 39(52.0) | 10(58.8) | 1.28(0.42-3.92) |  |
|  |  |  | A/A | 6(8.0) | 1(5.9) | 0.83(0.08-8.24) |  |
|  |  | **Dominant** | G/G | 30(40.0) | 6(35.3) | 1.00 | NS |
|  |  |  | A/G-A/A | 45(60.0) | 11(64.7) | 1.22(0.41-3.66) |  |
|  |  | **Recessive** | G/G-A/G | 69(92.0) | 16(94.1) | 1.00 | NS |
|  |  |  | A/A | 6(8.0) | 1(5.9) | 0.72(0.08-6.40) |  |
|  |  | **Overdominant** | G/G-A/A | 36(48.0) | 7(41.2) | 1.00 | NS |
|  |  |  | A/G | 39(52.0) | 10(58.8) | 1.32(0.45-3.83) |  |
|  |  | **Log-additive** | --- | --- | --- | 1.07(0.45-2.55) | NS |
| **IL12B**  **G/T** | **rs3212227** | **Codominant** | T/T | 48(64.0) | 9(52.9) | 1.00 | NS |
|  |  |  | T/G | 20(26.7) | 8(47.1) | 2.13(0.72-6.32) |  |
|  |  |  | G/G | 7(9.3) | 0(0.0) | 0.00(0.00-NA) |  |
|  |  | **Dominant** | T/T | 48(64.0) | 9(52.9) | 1.00 | NS |
|  |  |  | T/G-G/G | 27(36.0) | 8(47.1) | 1.58(0.55-4.57) |  |
|  |  | **Recessive** | T/T-T/G | 68(90.7) | 17(100.0) | 1.00 | NS |
|  |  |  | G/G | 7(9.3) | 0(0.0) | 0.00(0.00-NA) |  |
|  |  | **Overdominant** | T/T-G/G | 55(73.3) | 9(52.9) | 1.00 | NS |
|  |  |  | T/G | 20(26.7) | 8(47.1) | 2.44(0.83-7.21) |  |
|  |  | **Log-additive** | --- | --- | --- | 1.04(0.46-2.38) | NS |
| **IL28B**  **C/T** | **rs12979860** | **Codominant** | C/C | 34(45.3) | 7(41.2) | 1.00 | NS |
|  |  |  | T/C | 30(40.0) | 8(47.1) | 1.30(0.42-4.00) |  |
|  |  |  | T/T | 11(14.7) | 2(11.8) | 0.88(0.16-4.89) |  |
|  |  | **Dominant** | C/C | 34(45.3) | 7(41.2) | 1.00 | NS |
|  |  |  | T/C-T/T | 41(54.7) | 10(58.8) | 1.18(0.41-3.45) |  |
|  |  | **Recessive** | C/C-T/C | 64(85.3) | 15(88.2) | 1.00 | NS |
|  |  |  | T/T | 11(14.7) | 2(11.8) | 0.78(0.16-3.87) |  |
|  |  | **Overdominant** | C/C-T/T | 45(60.0) | 9(52.9) | 1.00 | NS |
|  |  |  | T/C | 30(40.0) | 8(47.1) | 1.33(0.46-3.84) |  |
|  |  | **Log-additive** | --- | --- | --- | 1.03(0.49-2.16) | NS |
| **CCL2**  **A/G** | **rs1024611** | **Codominant** | A/A | 40(53.3) | 10(58.8) | 1.00 | NS |
|  |  |  | G/A | 33(44.0) | 6(35.3) | 0.73(0.24-2.21) |  |
|  |  |  | G/G | 2(2.7) | 1(5.9) | 2.00(0.16-24.33) |  |
|  |  | **Dominant** | A/A | 40(53.3) | 10(58.8) | 1.00 | NS |
|  |  |  | G/A-G/G | 35(46.7) | 7(41.2) | 0.80(0.28-2.33) |  |
|  |  | **Recessive** | A/A-G/A | 73(97.3) | 16(94.1) | 1.00 | NS |
|  |  |  | G/G | 2(2.7) | 1(5.9) | 2.28(0.19-26.72) |  |
|  |  | **Overdominant** | A/A-G/G | 42(56.0) | 11(64.7) | 1.00 | NS |
|  |  |  | G/A | 33(44.0) | 6(35.3) | 0.69(0.23-2.07) |  |
|  |  | **Log-additive** | --- | --- | --- | 0.93(0.36-2.39) | NS |
| **DC-SIGN**  **A/G** | **rs735240** | **Codominant** | G/G | 27(36.0) | 8(47.1) | 1.00 | NS |
|  |  |  | G/A | 33(44.0) | 4(23.5) | 0.41(0.11-1.51) |  |
|  |  |  | A/A | 15(20.0) | 5(29.4) | 1.13(0.31-4.06) |  |
|  |  | **Dominant** | G/G | 27(36.0) | 8(47.1) | 1.00 | NS |
|  |  |  | G/A-A/A | 48(64.0) | 9(52.9) | 0.63(0.22-1.83) |  |
|  |  | **Recessive** | G/G-G/A | 60(80.0) | 12(70.6) | 1.00 | NS |
|  |  |  | A/A | 15(20.0) | 5(29.4) | 1.67(0.51-5.46) |  |
|  |  | **Overdominant** | G/G-A/A | 42(56.0) | 13(76.5) | 1.00 | NS |
|  |  |  | G/A | 33(44.0) | 4(23.5) | 0.39(0.12-1.31) |  |
|  |  | **Log-additive** | --- | --- | --- | 0.97(0.48-1.95) | NS |
| **TLR2**  **A/G** | **rs5743708** | **---** | G/G | 66(88.0) | 16(94.1) | 1.00 | NS |
|  |  |  | G/A | 9(12.0) | 1(5.9) | 0.46(0.05-3.88) |  |
| **TLR4**  **C/T** | **rs4986791** | **---** | C/C | 67(89.3) | 16(94.1) | 1.00 | NS |
|  |  |  | T/C | 8(10.7) | 1(5.9) | 0.52(0.06-4.49) |  |
| **TLR9**  **C/T** | **rs352140** | **Codominant** | T/T | 24(32.0) | 6(35.3) | 1.00 | NS |
|  |  |  | T/C | 38(50.7) | 9(52.9) | 0.95(0.30-3.00) |  |
|  |  |  | C/C | 13(17.3) | 2(11.8) | 0.62(0.11-3.50) |  |
|  |  | **Dominant** | T/T | 24(32.0) | 6(35.3) | 1.00 | NS |
|  |  |  | T/C-C/C | 51(68.0) | 11(64.7) | 0.86(0.29-2.61) |  |
|  |  | **Recessive** | T/T-T/C | 62(82.7) | 15(88.2) | 1.00 | NS |
|  |  |  | C/C | 13(17.3) | 2(11.8) | 0.64(0.13-3.12) |  |
|  |  | **Overdominant** | T/T-C/C | 37(49.3) | 8(47.1) | 1.00 | NS |
|  |  |  | T/C | 38(50.7) | 9(52.9) | 1.10(0.38-3.14) |  |
|  |  | **Log-additive** | --- | --- | --- | 0.82(0.38-1.80) | NS |

Data presented as number (%), OR, odds ratio; CI, confidence interval; NA, not applicable; NS, not significant (p-values above 0.05); IL, Interleukin; CCL 2, C-C motif chemokine ligand 2; DC-SIGN, dendritic cell-specific ICAM-grabbing non-integrin; TLR, Toll-like receptor.
^a^ SNP database (dbSNP) reference number (ID number).
^b^ p-value for comparison between infants without chorioretinitis and with chorioretinitis in cCMV group.

^c^ with chorioretinitis – if occurred at least in one eye.
