# Supplementary material for: Association between single nucleotide polymorphisms (SNPs) of IL1, IL12, IL28 and TLR4 and symptoms of congenital cytomegalovirus infection
Source: PLoS One. 2020 May 18;15(5):e0233096. doi: 10.1371/journal.pone.0233096 (PMC7233583; doi:10.1371/journal.pone.0233096)
Supplement: S8 Table — Data presented as number (%), OR, odds ratio; CI, confidence interval; NA, not applicable; NS, not significant (p-values above 0.05); IL, Interleukin; CCL 2, C-C motif chemokine ligand 2; DC-SIGN, dendritic cell-specific ICAM-grabbing non-integrin; TLR, Toll-like receptor. a SNP database (dbSNP) reference number (ID number). b P-value for comparison between infants without petechiae and with petechiae in cCMV group. (DOCX) [file pone.0233096.s008.docx]

**Table S8. Association between examined SNPs and petechiae.**

| **Gene** | **dbSNP IDnumber^a^** | **Genetic Model** | **Genotype** | **Without  petechiae n=77** | **With**  **petechiae n=15** | **OR (95% CI)** | **P-value^b^** |
| --- | --- | --- | --- | --- | --- | --- | --- |
| **IL1B**  **G/A** | **rs16944** | **Codominant** | G/G | 29(37.7) | 7(46.7) | 1.00 | NS |
|  |  |  | A/G | 41(53.2) | 8(53.3) | 0.81(0.26-2.48) |  |
|  |  |  | A/A | 7(9.1) | 0(0.0) | 0.00(0.00-NA) |  |
|  |  | **Dominant** | G/G | 29(37.7) | 7(46.7) | 1.00 | NS |
|  |  |  | A/G-A/A | 48(62.3) | 8(53.3) | 0.69(0.23-2.10) |  |
|  |  | **Recessive** | G/G-A/G | 70(90.9) | 15(100.0) | 1.00 | NS |
|  |  |  | A/A | 7(9.1) | 0(0.0) | 0.00(0.00-NA) |  |
|  |  | **Overdominant** | G/G-A/A | 36(46.8) | 7(46.7) | 1.00 | NS |
|  |  |  | A/G | 41(53.2) | 8(53.3) | 1.00(0.33-3.04) |  |
|  |  | **Log-additive** | --- | --- | --- | 0.60(0.23-1.56) | NS |
| **IL12B**  **G/T** | **rs3212227** | **Codominant** | T/T | 47(61.0) | 10(66.7) | 1.00 | NS |
|  |  |  | T/G | 24(31.2) | 4(26.7) | 0.78(0.22-2.76) |  |
|  |  |  | G/G | 6(7.8) | 1(6.7) | 0.78(0.08-7.24) |  |
|  |  | **Dominant** | T/T | 47(61.0) | 10(66.7) | 1.00 | NS |
|  |  |  | T/G-G/G | 30(39.0) | 5(33.3) | 0.78(0.24-2.52) |  |
|  |  | **Recessive** | T/T-T/G | 71(92.2) | 14(93.3) | 1.00 | NS |
|  |  |  | G/G | 6(7.8) | 1(6.7) | 0.85(0.09-7.58) |  |
|  |  | **Overdominant** | T/T-G/G | 53(68.8) | 11(73.3) | 1.00 | NS |
|  |  |  | T/G | 24(31.2) | 4(26.7) | 0.80(0.23-2.78) |  |
|  |  | **Log-additive** | --- | --- | --- | 0.84(0.34-2.09) | NS |
| **IL28B**  **C/T** | **rs12979860** | **Codominant** | C/C | 38(49.4) | 3(20.0) | 1.00 | NS |
|  |  |  | T/C | 26(33.8) | 12(80.0) | 5.85(1.50-22.78) |  |
|  |  |  | T/T | 13(16.9) | 0(0.0) | 0.00(0.00-NA) |  |
|  |  | **Dominant** | C/C | 38(49.4) | 3(20.0) | 1.00 | NS |
|  |  |  | T/C-T/T | 39(50.6) | 12(80.0) | 3.90(1.02-14.91) |  |
|  |  | **Recessive** | C/C-T/C | 64(83.1) | 15(100.0) | 1.00 | NS |
|  |  |  | T/T | 13(16.9) | 0(0.0) | 0.00(0.00-NA) |  |
|  |  | **Overdominant** | C/C-T/T | 51(66.2) | 3(20.0) | 1.00 | NS |
|  |  |  | T/C | 26(33.8) | 12(80.0) | 7.85(2.03-30.28) |  |
|  |  | **Log-additive** | --- | --- | --- | 1.28(0.59-2.76) | NS |
| **CCL2**  **A/G** | **rs1024611** | **Codominant** | A/A | 44(57.1) | 6(40.0) | 1.00 | NS |
|  |  |  | G/A | 31(40.3) | 8(53.3) | 1.89(0.60-6.00) |  |
|  |  |  | G/G | 2(2.6) | 1(6.7) | 3.67(0.29-46.84) |  |
|  |  | **Dominant** | A/A | 44(57.1) | 6(40.0) | 1.00 | NS |
|  |  |  | G/A-G/G | 33(42.9) | 9(60.0) | 2.00(0.65-6.17) |  |
|  |  | **Recessive** | A/A-G/A | 75(97.4) | 14(93.3) | 1.00 | NS |
|  |  |  | G/G | 2(2.6) | 1(6.7) | 2.68(0.23-31.58) |  |
|  |  | **Overdominant** | A/A-G/G | 46(59.7) | 7(46.7) | 1.00 | NS |
|  |  |  | G/A | 31(40.3) | 8(53.3) | 1.70(0.56-5.16) |  |
|  |  | **Log-additive** | --- | --- | --- | 1.90(0.73-4.94) | NS |
| **DC-SIGN**  **A/G** | **rs735240** | **Codominant** | G/G | 28(36.4) | 7(46.7) | 1.00 | NS |
|  |  |  | G/A | 34(44.2) | 3(20.0) | 0.35(0.08-1.49) |  |
|  |  |  | A/A | 15(19.5) | 5(33.3) | 1.33(0.36-4.93) |  |
|  |  | **Dominant** | G/G | 28(36.4) | 7(46.7) | 1.00 | NS |
|  |  |  | G/A-A/A | 49(63.6) | 8(53.3) | 0.65(0.21-1.99) |  |
|  |  | **Recessive** | G/G-G/A | 62(80.5) | 10(66.7) | 1.00 | NS |
|  |  |  | A/A | 15(19.5) | 5(33.3) | 2.07(0.61-6.95) |  |
|  |  | **Overdominant** | G/G-A/A | 43(55.8) | 12(80.0) | 1.00 | NS |
|  |  |  | G/A | 34(44.2) | 3(20.0) | 0.32(0.08-1.21) |  |
|  |  | **Log-additive** | --- | --- | --- | 1.06(0.51-2.21) | NS |
| **TLR2**  **A/G** | **rs5743708** | **---** | G/G | 69(89.6) | 13(86.7) | 1.00 | NS |
|  |  |  | G/A | 8(10.4) | 2(13.3) | 1.33(0.25-6.97) |  |
| **TLR4**  **C/T** | **rs4986791** | **---** | C/C | 70(90.9) | 13(86.7) | 1.00 | NS |
|  |  |  | T/C | 7(9.1) | 2(13.3) | 1.54(0.29-8.25) |  |
| **TLR9**  **C/T** | **rs352140** | **Codominant** | T/T | 24(31.2) | 6(40.0) | 1.00 | NS |
|  |  |  | T/C | 39(50.6) | 8(53.3) | 0.82(0.25-2.66) |  |
|  |  |  | C/C | 14(18.2) | 1(6.7) | 0.29(0.03-2.62) |  |
|  |  | **Dominant** | T/T | 24(31.2) | 6(40.0) | 1.00 | NS |
|  |  |  | T/C-C/C | 53(68.8) | 9(60.0) | 0.68(0.22-2.12) |  |
|  |  | **Recessive** | T/T-T/C | 63(81.8) | 14(93.3) | 1.00 | NS |
|  |  |  | C/C | 14(18.2) | 1(6.7) | 0.32(0.04-2.65) |  |
|  |  | **Overdominant** | T/T-C/C | 38(49.4) | 7(46.7) | 1.00 | NS |
|  |  |  | T/C | 39(50.6) | 8(53.3) | 1.11(0.37-3.37) |  |
|  |  | **Log-additive** | --- | --- | --- | 0.63(0.27-1.48) | NS |

Data presented as number (%), OR, odds ratio; CI, confidence interval; NA, not applicable; NS, not significant (p-values above 0.05); IL, Interleukin; CCL 2, C-C motif chemokine ligand 2; DC-SIGN, dendritic cell-specific ICAM-grabbing non-integrin; TLR, Toll-like receptor.
^a^ SNP database (dbSNP) reference number (ID number).

^b^ p-value for comparison between infants without petechiae and with petechiae in cCMV group.
