# Supplementary material for: Association between single nucleotide polymorphisms (SNPs) of IL1, IL12, IL28 and TLR4 and symptoms of congenital cytomegalovirus infection
Source: PLoS One. 2020 May 18;15(5):e0233096. doi: 10.1371/journal.pone.0233096 (PMC7233583; doi:10.1371/journal.pone.0233096)
Supplement: S9 Table — Data presented as number (%), OR, odds ratio; CI, confidence interval; NA, not applicable; NS, not significant (p-values above 0.05); IL, Interleukin; CCL 2, C-C motif chemokine ligand 2; DC-SIGN, dendritic cell-specific ICAM-grabbing non-integrin; TLR, Toll-like receptor. a SNP database (dbSNP) reference number (ID number). b P-value for comparison between infants without hepatomegaly and with hepatomegaly in cCMV group. (DOCX) [file pone.0233096.s009.docx]

**Table S9. Association between examined SNPs and hepatomegaly.**

| **Gene** | **dbSNP IDnumber^a^** | **Genetic Model** | **Genotype** | **Without hepatomegaly n=77** | **With**  **hepatomegaly n=15** | **OR (95% CI)** | **P-value^b^** |
| --- | --- | --- | --- | --- | --- | --- | --- |
| **IL1B**  **G/A** | **rs16944** | **Codominant** | G/G | 27(35.1) | 9(60.0) | 1.00 | NS |
|  |  |  | A/G | 43(55.8) | 6(40.0) | 0.42(0.13-1.31) |  |
|  |  |  | A/A | 7(9.1) | 0(0.0) | 0.00(0.00-NA) |  |
|  |  | **Dominant** | G/G | 27(35.1) | 9(60.0) | 1.00 | NS |
|  |  |  | A/G-A/A | 50(64.9) | 6(40.0) | 0.36(0.12-1.12) |  |
|  |  | **Recessive** | G/G-A/G | 70(90.9) | 15(100.0) | 1.00 | NS |
|  |  |  | A/A | 7(9.1) | 0(0.0) | 0.00(0.00-NA) |  |
|  |  | **Overdominant** | G/G-A/A | 34(44.2) | 9(60.0) | 1.00 | NS |
|  |  |  | A/G | 43(55.8) | 6(40.0) | 0.53(0.17-1.63) |  |
|  |  | **Log-additive** | --- | --- | --- | 0.36(0.13-1.01) | NS |
| **IL12B**  **G/T** | **rs3212227** | **Codominant** | T/T | 47(61.0) | 10(66.7) | 1.00 | NS |
|  |  |  | T/G | 23(29.9) | 5(33.3) | 1.02(0.31-3.34) |  |
|  |  |  | G/G | 7(9.1) | 0(0.0) | 0.00(0.00-NA) |  |
|  |  | **Dominant** | T/T | 47(61.0) | 10(66.7) | 1.00 | NS |
|  |  |  | T/G-G/G | 30(39.0) | 5(33.3) | 0.78(0.24-2.52) |  |
|  |  | **Recessive** | T/T-T/G | 70(90.9) | 15(100.0) | 1.00 | NS |
|  |  |  | G/G | 7(9.1) | 0(0.0) | 0.00(0.00-NA) |  |
|  |  | **Overdominant** | T/T-G/G | 54(70.1) | 10(66.7) | 1.00 | NS |
|  |  |  | T/G | 23(29.9) | 5(33.3) | 1.17(0.36-3.82) |  |
|  |  | **Log-additive** | --- | --- | --- | 0.66(0.25-1.77) | NS |
| **IL28B**  **C/T** | **rs12979860** | **Codominant** | C/C | 37(48.0) | 4(26.7) | 1.00 | NS |
|  |  |  | T/C | 30(39.0) | 8(53.3) | 2.47(0.68-8.99) |  |
|  |  |  | T/T | 10(13.0) | 3(20.0) | 2.77(0.53-14.48) |  |
|  |  | **Dominant** | C/C | 37(48.0) | 4(26.7) | 1.00 | NS |
|  |  |  | T/C-T/T | 40(52.0) | 11(73.3) | 2.54(0.74-8.69) |  |
|  |  | **Recessive** | C/C-T/C | 67(87.0) | 12(80.0) | 1.00 | NS |
|  |  |  | T/T | 10(13.0) | 3(20.0) | 1.68(0.40-6.99) |  |
|  |  | **Overdominant** | C/C-T/T | 47(61.0) | 7(46.7) | 1.00 | NS |
|  |  |  | T/C | 30(39.0) | 8(53.3) | 1.79(0.59-5.45) |  |
|  |  | **Log-additive** | --- | --- | --- | 1.74(0.81-3.75) | NS |
| **CCL2**  **A/G** | **rs1024611** | **Codominant** | A/A | 43(55.8) | 7(46.7) | 1.00 | NS |
|  |  |  | G/A | 32(41.6) | 7(46.7) | 1.34(0.43-4.22) |  |
|  |  |  | G/G | 2(2.6) | 1(6.7) | 3.07(0.24-38.55) |  |
|  |  | **Dominant** | A/A | 43(55.8) | 7(46.7) | 1.00 | NS |
|  |  |  | G/A-G/G | 34(44.2) | 8(53.3) | 1.45(0.48-4.38) |  |
|  |  | **Recessive** | A/A-G/A | 75(97.4) | 14(93.3) | 1.00 | NS |
|  |  |  | G/G | 2(2.6) | 1(6.7) | 2.68(0.23-31.58) |  |
|  |  | **Overdominant** | A/A-G/G | 45(58.4) | 8(53.3) | 1.00 | NS |
|  |  |  | G/A | 32(41.6) | (746.7) | 1.23(0.41-3.74) |  |
|  |  | **Log-additive** | --- | --- | --- | 1.50(0.58-3.90) | NS |
| **DC-SIGN**  **A/G** | **rs735240** | **Codominant** | G/G | 31(40.3) | 4(26.7) | 1.00 | NS |
|  |  |  | G/A | 29(37.7) | 8(53.3) | 2.14(0.58-7.87) |  |
|  |  |  | A/A | 17(22.1) | 3(20.0) | 1.37(0.27-6.84) |  |
|  |  | **Dominant** | G/G | 31(40.3) | 4(26.7) | 1.00 | NS |
|  |  |  | G/A-A/A | 46(59.7) | 11(73.3) | 1.85(0.54-6.35) |  |
|  |  | **Recessive** | G/G-G/A | 60(77.9) | 12(80.0) | 1.00 | NS |
|  |  |  | A/A | 17(22.1) | 3(20.0) | 0.88(0.22-3.49) |  |
|  |  | **Overdominant** | G/G-A/A | 48(62.3) | 7(46.7) | 1.00 | NS |
|  |  |  | G/A | 29(37.7) | 8(53.3) | 1.89(0.62-5.76) |  |
|  |  | **Log-additive** | --- | --- | --- | 1.22(0.59-2.52) | NS |
| **TLR2**  **A/G** | **rs5743708** | **---** | G/G | 68(88.3) | 14(93.3) | 1.00 | NS |
|  |  |  | G/A | 9(11.7) | 1(6.7) | 0.54(0.06-4.61) |  |
| **TLR4**  **C/T** | **rs4986791** | **---** | C/C | 71(92.2) | 12(80.0) | 1.00 | NS |
|  |  |  | T/C | 6(7.8) | 3(20.0) | 2.96(0.65-13.46) |  |
| **TLR9**  **C/T** | **rs352140** | **Codominant** | T/T | 26(33.8) | 4(26.7) | 1.00 | NS |
|  |  |  | T/C | 39(50.6) | 8(53.3) | 1.33(0.36-4.89) |  |
|  |  |  | C/C | 12(15.6) | 3(20.0) | 1.62(0.31-8.43) |  |
|  |  | **Dominant** | T/T | 26(33.8) | 4(26.7) | 1.00 | NS |
|  |  |  | T/C-C/C | 51(66.2) | 11(73.3) | 1.40(0.41-4.84) |  |
|  |  | **Recessive** | T/T-T/C | 65(84.4) | 12(80.0) | 1.00 | NS |
|  |  |  | C/C | 12(15.6) | 3(20.0) | 1.35(0.33-5.53) |  |
|  |  | **Overdominant** | T/T-C/C | 38(49.4) | 7(46.7) | 1.00 | NS |
|  |  |  | T/C | 39(50.6) | 8(53.3) | 1.11(0.37-3.37) |  |
|  |  | **Log-additive** | --- | --- | --- | 1.28(0.57-2.88) | NS |

Data presented as number (%), OR, odds ratio; CI, confidence interval; NA, not applicable; NS, not significant (p-values above 0.05); IL ,Interleukin; CCL2, C-C motif chemokine ligand 2; DC-SIGN, dendritic cell-specific ICAM-grabbing non-integrin; TLR, Toll-like receptor.
^a^ SNP database (dbSNP) reference number (ID number).

^b^ P-value for comparison between infants without hepatomegaly and with hepatomegaly in cCMV group.
