# Supplementary material for: Association between single nucleotide polymorphisms (SNPs) of IL1, IL12, IL28 and TLR4 and symptoms of congenital cytomegalovirus infection
Source: PLoS One. 2020 May 18;15(5):e0233096. doi: 10.1371/journal.pone.0233096 (PMC7233583; doi:10.1371/journal.pone.0233096)
Supplement: Attachment — Submitted filename: Response to Reviewers1.docx [file pone.0233096.s010.docx]

We are pleased to resubmit the revised version of our manuscript PONE-D-20-04592 entitled „Association between single nucleotide polymorphisms (SNPs) of IL1, IL12, IL28 and TLR4 and symptoms of congenital cytomegalovirus infection”.

We would like to thank the reviewers for their constructive comments. We have introduced changes according to the reviewers’ comments. All changes are highlighted in yellow in marked-up version of the article.

Please find our response to the reviewers’ comments, below:

**Reviewer #1: The manuscript topic is an outdated approach, where the variants in the known immune genes are associated with the CMV infection. These genes were previously implied in numerous studies, and replicated poorly, suggesting lack of real significance or at the least more complicated situation that previously assumed. Also, the focus of the clinically manifest disease will likely depend on the host genes, causing more selection bias in the study design. Lack of significance and traces of related phenotypes support this, and suggest an accidental finding (due to lack of multiple-testing adjustment, which is mandatory due to various genetic models being used in the analysis).** **The main hypothesis of this study should not be so focused on CMV itself (since the vast majority of people are positive), but to the underlying genetic characteristics that predispose some of these infected people/neonates towards infection. This is likely to be achieved through meta-analytic study. The current study is substantially underpowered, and hence of very limited contribution.**

Reply: We thank the reviewer for this important comment. Our study was single-centre and prospective one, conducted between 2016 and 2019 in homogenous population of Caucasian newborns. The methodology has been chosen based on current trends in the field of SNP associations with infectious diseases. Both hypothesis-driven candidate gene approach and genome-wide associations are essentially gene association studies as well as both are subject to the same artefacts of spurious association findings. In our study the former approach was chosen based on our previous expertise in the field and available resources. We used customized TaqMan Assay (rather than RLFP-PCR) which provide accurate and reproductive results. We acknowledged that the power of association study is improved if the sampling strategy takes account of exposure heterogeneity, though this is not necessarily easy to do, especially in the setting of congenital CMV infection. We also acknowledge the lack of correction for multi testing and these limitations were mentioned in the manuscript.

We appreciate the reviewer’s point of view that the main hypothesis of this study should not be so focused on CMV itself (since the vast majority of people are positive). We agree that the vast majority of people are positive. The prevalence of CMV seropositive women in reproductive age is about 80% in Poland _(Siennicka_ High Seroprevalence of CMV Among Women of Childbearing Age Implicates High Burden of Congenital Cytomegalovirus Infection in Poland_ Polisch J Microbiol 2016)._ The prevalence of seropositive newborns would be similar - most of newborn infants are seropositive due to transplacental transfer of maternal IgG but not to congenital infection. Nevertheless, a rate of cCMV is only about 0,5 % (in Poland estimated rate is 2,2 to 3,7%). Additionally, immaturity of fetal immune system makes this population very unique and may influence on the role of immune genes variations in congenital infection. Therefore we decided to examine the association between SNPs in genes encoding selected cytokines and cytokine receptors in newborns (also preterms) with proven congenital CMV infection. We agree that these genes were previously implied in numerous studies, and replicated poorly, however most of them were not performed in newborn infants, and if they were performed, the study populations were comparable or even smaller than ours. We underlined the need for further studies in larger population in our manuscript. We also agree that meta-analysis incorporating published SNP genotyping data is warranted to evaluate factors which predispose neonates towards CMV infection, thus data collected in studies like ours is desirable.

**Reviewer #2:**

**1. Table 1: summation of cCMV group (n=49) and control group (n=70) should be 119 not 129 for Cesarean section**

Reply: We would like to thank the reviewer for attention to details. We have addressed this comment by correcting data in Table 1.

**2. In all tables the foot notes should clearly state to which groups’ statistical comparisons the P values belong to. (i.e. cCMV and control groups)**

Reply: We have followed reviewer’s recommendation and we are very grateful for this suggestion. We have added suitable information in the foot notes to state which groups’ statistical comparison were presented. We have presented that information by using “b” superscript in all footnotes.

**3. For tables where SNP genotypes are presented, for each SNP the change should be given. For example rs16944 (G/A)**

Reply: We would like to thank the reviewer for attention to details. We have supplemented the tables with information about the change in nucleotides for each SNP gene.

**4. Either in the Materials or in Introduction section why these candidate genes was chosen should be briefly explained (i.e. cytokines X,Y,Z were chosen for their pro-inflammatory role, etc.. ). This will also make the manuscript look more hypothesis driven.**

Reply: In the Material and Methods section, we have added additional section called “Candidate genes selection”, where we provided rationale for selecting individual SNPs for the study. We have supported it with additional appropriate references.

**5. AIC values presented in table 5 doesn’t convey much information as the reader doesn’t know the AIC values for other alternate models / effects of other not presented SNPs on a specific symptom.**

Reply: We appreciate the reviewer’s precise analysis of the presented data. We understand that our presentation of data within Table 5 was not adequately detailed. Following the reviewer’s comments, we have made all the data underlying our findings fully available for the reader. Thus, we have moved four tables concerning symptoms with statistical significant findings from the Supplementary File to the main article. We have created four separate tables (Table 5-8) for each symptom instead of combined Table 5 from the original version of the article.

In all new Tables, AIC values have been added, in order to allow readers to see the results of other alternate models. The best fitted model based on AIC for each SNP have been presented by using “ c” superscript in footnotes in all four new Tables (5 to 8).

As the supporting information, we left nine tables (Tables S1-9) concerning data associations between examined SNPs and other analyzed symptoms among cCMV group, where no statistical finding were observed.

We hope, that new presentation of our results is now more informative for the reader.

**6. Data on HCMV viral load, and maternal genotypes would also be interesting to examine.**

As far as HCMV viral load is concerned, we have put these data into separate article which is now being reviewed in other medical journal.

We agree that maternal genotypes would be interesting to explore but our current project was not designed to address those issues. This was dictated by the fact that the present study was a single-center and conducted in hospital with no maternity unit. However, we strongly agree that it would be worth to incorporate maternal genotypes into a new multicenter project in the future.

**7. Thorough language, word usage, spelling check is advised.**

We have proofread the paper to eliminate all languages spelling errors.
