# Supplementary material for: Association between single nucleotide polymorphisms (SNPs) of IL1, IL12, IL28 and TLR4 and symptoms of congenital cytomegalovirus infection
Source: PLoS One. 2020 May 18;15(5):e0233096. doi: 10.1371/journal.pone.0233096 (PMC7233583; doi:10.1371/journal.pone.0233096)
Supplement: Attachment — Submitted filename: Response to Reviewers_2.docx [file pone.0233096.s011.docx]

We are pleased to resubmit the revised version of our manuscript PONE-D-20-04592R1 entitled „Association between single nucleotide polymorphisms (SNPs) of IL1, IL12, IL28 and TLR4 and symptoms of congenital cytomegalovirus infection”.

We would like to thank the reviewers for their constructive comments. We have tried to introduced changes according to the reviewers’ comments. Please find our response to the reviewers’ comments, below:

**Reviewer #1: Thank you; you have not provided responses to all of my statements; please, at the very least address some of these issues in the limitations section**

**Reply:** We thank the Reviewer for this comment to expand the limitation section in our article. Now, we hope that constructive remarks in this section make the article much more valuable for the readers and researchers. The improved limitation section is as below:

“The present study has also some limitations. We are aware that hypothesis-driven candidate gene approach is essentially gene association study, however, it is also a subject to the artefacts of spurious association findings. Genes examined in the current study had been previously implied and some of them had been replicated poorly. However, most of these genes were not performed in newborn infants or if they were performed in that age group, the study populations were comparable or even smaller than ours.

What is more, it seems that different genes may take part in predisposition to the disease, while others host genes might influence on the clinical manifestation of the disease. We admit that the situation might be more complicated than it was previously assumed. Moreover, there are plenty of other external factors that could impact on the results: different HCMV strains, primary or secondary maternal infection, time of the fetus infection (which trimester). However, the current study was not designed to address these issues.

We acknowledge that the power of association study is improved if the sampling strategy takes account of exposure heterogenity, though this is not necessarily easy to do, especially in the setting of cCMV infection. In addition, no correction for multiple testing was applied, and so type I error cannot be completely ruled out.

Nevertheless, our study group of 92 cCMV-infected newborns is larger than in our previous research, we are aware that the study sample size might be still not large enough as for genetic studies. Undoubtedly, future studies on cCMV are demanded to replicate above-mentioned findings in a much larger cohort. Moreover, there is a need for other studies of genetic characteristics of the unique and immature fetus-host and its predisposition to the infection, not only limiting to HCMV itself. We underline the necessity for meta-analytic studies in this subject. “
